# Emerging Treatment Options for Gastroenteropancreatic Neuroendocrine Tumors

**DOI:** 10.3390/jcm9113655

**Published:** 2020-11-13

**Authors:** Mauro Cives, Eleonora Pelle’, Jonathan Strosberg

**Affiliations:** 1 Department of Biomedical Sciences and Human Oncology, University of Bari, 70124 Bari, Italy; mauro.cives@uniba.it (M.C.); Eleonora.pelle89@gmail.com (E.P.); 2 IRCCS Istituto Tumori Giovanni Paolo II, 70124 Bari, Italy; 3 Department of Gastrointestinal Oncology, H. Lee Moffitt Cancer Center and Research Institute, Tampa, FL 33612, USA

**Keywords:** neuroendocrine neoplasms, neuroendocrine tumors, neuroendocrine carcinomas, surufatinib, lenvatinib, axitinib, immunotherapy, peptide receptor radionuclide therapy (PRRT)

## Abstract

Treatment options for neuroendocrine tumors (NETs) and carcinomas (NECs) are expanding. Early-phase studies have shown preliminary evidence of the antitumor activity of alpha-emitting peptide receptor radionuclide therapy (PRRT), and novel radiopeptides incorporating somatostatin receptor antagonists (rather than agonists) have been developed. Several tyrosine kinase inhibitors (TKIs) with antiangiogenic potential have been evaluated in patients with NETs, including lenvatinib, axitinib, cabozantinib and pazopanib. Recently, two phase 3 clinical trials have demonstrated the efficacy and safety of surufatinib, an inhibitor of vascular endothelial growth factor receptor (VEGFR)-1, -2, -3, fibroblast growth factor receptor (FGFR)-1 and colony stimulating factor-1 receptor (CSF-1R), in patients with pancreatic and extra-pancreatic NETs. Multiple clinical trials of combination immunotherapy have been recently completed, but interpretation of the results is hampered by small samples sizes and discordant outcomes. This review summarizes recent data on emerging treatments for neuroendocrine neoplasms.

## 1. Introduction

Neuroendocrine tumors (NETs) are heterogeneous neoplasms arising in secretory cells of the diffuse neuroendocrine system. They are characterized by a relatively indolent growth and the ability to secrete biogenic amines and peptide hormones [1]. Gastroenteropancreatic NETs (GEP-NETs) include NETs of the gastrointestinal tract (GI-NETs) and pancreatic NETs (panNETs), and can be subdivided in well-differentiated (low-, intermediate- or high-grade) tumors and poorly differentiated carcinomas (NECs), according to their morphology and proliferative activity [2].

Recent years have seen a surge of research on treatments for advanced GEP-NETs, and the therapeutic landscape of these malignancies has expanded considerably. Somatostatin analogs (SSAs) have demonstrated both antisecretory and antitumor efficacy [3,4]. Peptide receptor radionuclide therapy (PRRT) with ^177^Lutetium (Lu)-DOTATATE was associated with significant prolongation of progression-free survival (PFS) in patients with progressive midgut NETs [5,6]. The mTOR inhibitor everolimus has shown antitumor activity against NETs regardless of their primary site [7,8], and the tyrosine kinase inhibitor (TKI) sunitinib has been approved for panNETs [9]. The clinical benefit of chemotherapy with temozolomide and capecitabine has been recently demonstrated in a randomized trial [10], and multiple retrospective series [11] have reported the efficacy of liver-directed therapies for controlling tumor growth. However, despite these advances, metastatic NETs remain incurable and new effective treatments are warranted.

Several clinical trials are currently enrolling NET patients. Given the pivotal role of somatostatin receptors (SSTRs) in NET biology and the impressive results observed with ^177^Lu-DOTATATE, innovative PRRT strategies encompassing the use of alpha-emitting radiolabeled somatostatin analogs (SSAs) or radiolabeled SSTR antagonists are being actively investigated. A variety of new TKIs with antiangiogenic properties are also being tested to target the aberrant vascularization of NETs. Multiple clinical trials of immunotherapeutic agents, alone or in combination, are underway to evaluate the role of immune checkpoint blockade in NETs. In this review, we summarize the most recent advances of clinical research in the NET field, focusing on the clinical efficacy and toxicity of new emerging systemic treatments for patients with advanced GEP-NETs.

## 2. Innovative PRRT Strategies

PRRT is a form of systemic radiotherapy that allows targeted delivery of radionuclides to tumor cells expressing high levels of SSTRs [12]. For many years, evidence of the antitumor activity of PRRT in patients with NETs was derived only from early phase studies using SSAs radiolabeled with ^90^Yttrium (^90^Y) or ^177^Lu, but the randomized, phase 3 NETTER-1 trial [5] has recently provided high-level evidence of the activity and safety of this form of treatment. The NETTER-1 trial investigated ^177^Lu-DOTATATE versus high-dose octreotide long-acting repeatable (LAR) (60 mg/28 days) in patients with advanced, OctreoScan-positive midgut NETs who progressed on standard-dose octreotide LAR. After a median follow-up of 14 months, ^177^Lu-DOTATATE resulted in a 79% reduction in risk of progression or death compared with the control group (*p* < 0.0001; hazard ratio: 0.21; 95% confidence interval (CI), 0.13–0.33).

Radiolabeled peptides used for PRRT consist of a radionuclide isotope, a carrier molecule (generally derived from the SSA octreotide), and a chelator that binds them stabilizing the resulting complex [12]. Innovative PRRT radiopeptides incorporate different radionuclides or different carriers, and their clinical development is underway. The most relevant characteristics of a radioisotope are the path length and the linear energy transfer (LET). Although a longer path length may be useful for treating large volume tumors, damage to surrounding healthy tissue may occur. The LET measures the ionizing density and, hence, the molecular damage of a particle per unit length. Particles with high LET provide more severe and less reparable cell damage than those with low LET [13]. Both ^177^Lu and ^90^Y are β-emitting particles, meaning that they release negatively charged electrons trough the β-decay process. These particles are characterized by relatively long path length (up to 12 mm) and low LET (0.2 keV/µm), thus producing single strand DNA damage which is influenced by the cell cycle phase [14]. To maximize the therapeutic effects of PRRT while reducing its off-tumor toxicities, α-emitters have been developed. The α-emitters release positively charged particles (two neutrons and two protons) through the alpha decay process. These particles have a high LET and a short-range (between 40 and 100 μm), resulting in severe DNA damage irrespective of the cell cycle phase and oxygen concentration, with minimal radiotoxicity to the surrounding tissue [15].

Among emerging α-emitters, ^225^Ac-DOTATATE, ^213^Bi-DOTATOC and ^212^Pb-DOTAMTATE have shown promising results in early clinical studies. ^225^Actinium (^225^Ac) is a pure α-emitter with a half-life of 10 days. In a first-in-human study, ^225^Ac was tested in 10 patients with NETs progressing after β-emitting PRRT, with evidence of safety and tolerability [16]. A subsequent study prospectively investigated ^225^Ac-DOTATATE in 32 patients with SSTR-positive GEP-NET who received at least two prior lines of systemic treatment including ^177^Lu-DOTATATE [17]. The treatment schedule consisted of 100 kBq (2.7 μCi)/kg of ^255^Ac-DOTATATE at 8-week intervals up to a cumulative dose of 55,500 kBq (1.5 mCi). After a median follow-up of 8 months, there were no deaths or progressive events in the 24 patients assessable for response. Among them, 15 patients exhibited a partial response and 9 stable disease by Response Evaluation Criteria in Solid Tumors (RECIST) 1.1. The most common adverse events associated with the investigational treatment were loss of appetite, nausea and vomiting. These toxicities may have been related to the amino acid infusion rather than the treatment itself, as commonly observed in patients receiving β-emitters.

^213^Bismuth (^213^Bi) is a mixed α/β-emitter with a half-life of 46 min. In a first-in-human study [18] enrolling 7 patients with NET liver metastases progressing on treatment with ^90^Y/^177^Lu-DOTATOC, the intra-arterial administration of ^213^Bi-DOTATOC into the hepatic artery produced one complete response, two partial responses and three stable diseases according to RECIST criteria. The side effects of ^213^Bi-DOTATOC included moderate chronic kidney toxicity and mild acute hematologic toxicity. ^212^Lead (^212^Pb) emits α particles of potential therapeutic interest following its decay to sTable 2^08^Pb. In murine models of NETs, a combination of 5-flurouracil and ^212^Pb-DOTAMTATE induced complete responses in approximately 80% of the tested animals [19]. On this basis, a phase 1 dose-escalation study of ^212^Pb-DOTAMTATE has been initiated with a target accrual of 50 patients with advanced SSTR-positive NETs (NCT03466216). In a preliminary analysis of 16 treated patients, ^212^Pb-DOTAMTATE demonstrated a favorable safety profile. Among six patients who received the highest dose escalation, the objective response rate (ORR) was 83%, with one complete response and five partial responses (three of them classified as near complete responses) [20].

Despite minimal or no internalization of the antagonist-receptor complex into tumor cells, SSTR antagonists may have several advantages as compared with SSTR agonists in the design of PRRT radiopeptides. First, antagonists may bind SSTRs in both their active and inactive conformations, thus occupying more binding sites than agonists. Second, antagonists show a lower dissociation rate than agonists. As result, antagonists tend to show higher tumor uptake and higher tumor retention when compared to SSTR agonists [21]. ^177^Lu-DOTA-JR11, also named ^177^Lu-Satoreotide Tetraxetan, is a radiolabeled SSTR antagonist and has been tested in a phase 1 trial [22] of 20 patients with well-differentiated, SSTR-positive, heavily pre-treated NET. Patients first underwent a dosimetry study to determine the therapeutic activity that could be safely administered. Then, they received this activity split into two equal cycles delivered 3 months apart. Overall, six patients received one cycle of ^177^Lu-DOTA-JR11, while two cycles were administered in the remaining 14 patients. Grade 4 hematologic toxicities occurred in four out of the seven patients who first received ^177^Lu-DOTA-JR11, probably as result of the high affinity of the radiopeptide to SSTRs expressed within the bone marrow. The study protocol was therefore modified to limit the cumulative absorbed bone marrow dose to 1 Gy. Overall, ^177^Lu-DOTA-JR11 resulted in an ORR of 45% (5% complete response; 40% partial response), with stable disease in the 40% of the cohort. A median PFS of 21 months was reported. Another phase 1/2 study has recently assessed the safety of different dosages of ^177^Lu-DOTA-JR11 in a total of 35 patients with progressive, low-to-intermediate grade NETs [23]. Grade 3 or worse treatment-related hematologic adverse events were observed in 12 patients (34%), while treatment discontinuation was reported in 6 patients. The disease control rate (DCR) at 12 months was 90%. Table 1 summarizes the efficacy results of clinical trials evaluating alpha-emitters or somatostatin-receptor antagonists in patients with NETs.

## 3. New Antiangiogenic Agents

NETs are among the most vascularized cancers, with an intratumoral vessel density 10-fold higher as compared with many other carcinomas [24]. This is not particularly surprising, as a high vascular supply is required for the physiologic functions of normal endocrine tissue. As result of the aberrant activation of the hypoxia-inducible factor-1α (HIF-1α) transcriptional program, NETs overexpress proangiogenic factors including vascular endothelial growth factor (VEGF), fibroblast growth factor (FGF), platelet-derived growth factor (PDGF), semaphorins and angiopoietins, as well as their cognate receptors [24]. Multiple TKIs with antiangiogenic properties are currently under clinical investigation in patients with advanced GEP-NETs. Among them, surufatinib, lenvatinib, axitinib, cabozantinib and pazopanib have shown promising efficacy in phase 2 or 3 clinical trials.

**Figure 1:**
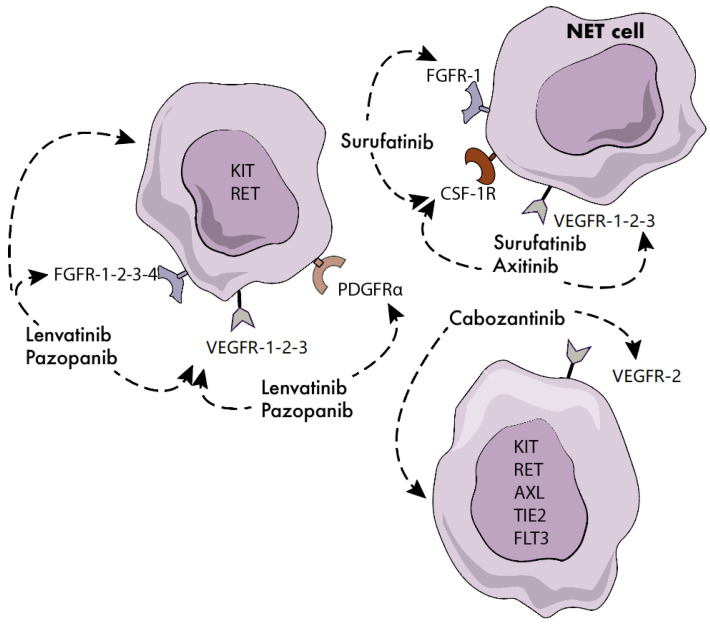
Molecular targets of novel tyrosine kinase inhibitors (TKIs) in neuroendocrine tumors (NETs). FGFR: fibroblast growth factor receptor; CSF-1R: colony stimulating factor 1 receptor; VEGFR: vascular endothelial growth factor receptor; PDGFR: platelet-derived growth factor.

Surufatinib is an orally active, potent, selective inhibitor of vascular endothelial growth factor receptor (VEGFR)-1, -2, -3, fibroblast growth factor receptor (FGFR)-1 and colony stimulating factor-1 receptor (CSF-1R) (Figure 1). Since the activation of FGFR-1 and CSF-1R has been described as one of the main determinants of acquired resistance to VEGFR inhibitors, surufatinib has the potential to overcome resistance to first-generation TKIs including sunitinib [25]. Surufatinib has been tested at a dosage of 300 mg once daily in a single-arm, multicenter, phase 1b/2 trial of 81 patients with low-to-intermediate grade advanced NETs [26]. A median PFS of 21.2 months and 13.4 months was reported in 42 patients with panNETs and 39 patients with extrapancreatic NETs, respectively. The ORR was 19% and 15% in the pancreatic and extrapancreatic NET cohorts, and hypertension, proteinuria, hyperuricemia, hypertriglyceridemia and diarrhea were the most frequent treatment-related grade 3 or worse adverse events. Based on these results, two randomized, double-blind, placebo-controlled, phase 3 studies recently investigated the safety and efficacy of surufatinib in Chinese patients with well-differentiated, progressive, advanced pancreatic (SANET-p trial) and extrapancreatic NETs (SANET-ep trial). Both trials have been terminated at their pre-planned interim analysis after meeting the primary endpoint of improved PFS. The SANET-ep trial [27] randomized 198 patients with extrapancreatic NETs to receive surufatinib or placebo in a 2:1 ratio. The majority of the enrolled patients (84%) had G2 tumors. The investigator-assessed median PFS was 9.2 vs. 3.8 months in the surufatinib and placebo arms respectively (hazard ratio (HR) = 0.33, 95% CI 0.22–0.5; *p* < 0.0001). The results of the SANET-p trial [28] have been recently disclosed. The study randomized 172 patients with panNETs to receive surufatinib or placebo in a 2:1 ratio. The investigator-assessed median PFS were 10.9 and 3.7 months in the surufatinib and placebo arm respectively (HR: 0.49, 95% CI 0.32–0.75; *p* = 0.001), in the presence of an investigator-assessed ORR of 19% in the investigational arm. It remains unclear whether the therapeutic index of surufatinib is substantially improved compared to the TKI sunitinib, which is already approved for panNETs, or whether surufatinib is active in patients who are refractory to sunitinib.

Lenvatinib is an oral TKI that selectively targets VEGFR-1, -2, -3, FGFR-1, -2, -3, -4, platelet-derived growth factor receptor α (PDGFRα), KIT and RET. The drug has been approved by the Food and Drug Administration (FDA) for patients with differentiated thyroid cancer, renal cell carcinoma, hepatocellular carcinoma and endometrial cancer. The phase 2 TALENT study [29] investigated lenvatinib 24 mg daily in 55 patients with panNETs and 56 patients with gastrointestinal NETs. All patients had progressive disease according to RECIST criteria, and prior therapy with targeted agents was mandatory for enrollment in the panNET cohort. By central radiology review, an ORR of 42% and 16% has been preliminarily reported in the panNET and gastrointestinal NET cohorts, respectively. After a median follow-up of 19 months, the median PFS was 15 months for either patient cohort. Hypertension, fatigue and diarrhea were the most frequent G3/4 treatment-emergent adverse events, with 10% of patients discontinuing the treatment due to toxicity.

Axitinib is a TKI with picomolar potency against VEGFR-1, -2 and -3. Such an inhibitory potency is up to 450 times higher than that of first-generation TKIs targeting VEGFRs. At present, the drug is approved for the treatment of advanced renal cell carcinoma. An open-label, phase 2 study [30] investigated axitinib 5 mg twice daily in 30 patients with progressive, advanced, low-to-intermediate grade NET of extra-pancreatic origin. After a median follow-up of 29 months, a median PFS of 27 months was reported. Grade 3/4 hypertension was recorded in the 63% of the cohort, leading to treatment discontinuation in one fifth of enrolled patients. The double-blind, phase 2/3 AXINET trial (NCT01744249) has randomized 255 patients with advanced, low-to-intermediate grade, progressive, non-pancreatic NETs to receive axitinib plus octreotide LAR or placebo plus octreotide LAR. The study results are still pending, and their release is expected in the next few months.

Cabozantinib is an orally active, potent inhibitor of MET, VEGFR2, KIT, RET, AXL, TIE2 and FLT3. Based on preclinical findings showing the ability of the drug to inhibit the viability and migration of NET cells [31], cabozantinib was investigated in a phase 2 study [32] enrolling 20 patients with panNETs and 41 patients with non-pancreatic NETs. All patients had well-differentiated tumors and progressive disease according to RECIST 1.1 criteria. Based on preliminary results, the drug was associated with an ORR of 15% in either cohort. A median PFS of 22 and 31 months was recorded in the pancreatic and non-pancreatic subgroup respectively. Grade 3/4 toxicities included hypertension, hypophosphatemia, diarrhea, lymphopenia, thrombocytopenia and fatigue. The phase 3 CABINET trial is currently underway in the US to assess the efficacy of cabozantinib 60 mg daily in patients with advanced progressive NET (NCT03375320).

Pazopanib is an oral TKI targeting VEGFR -1, -2, -3, FGFR-1, -3, -4, PDGFR-α and -β and c-KIT. The multi-center, open-label, phase 2 PAZONET trial [33] evaluated pazopanib in 44 patients with advanced, progressive, well-differentiated NETs. The study documented a median PFS of 9 months, with a clinical benefit rate (complete response + partial response + stable disease by RECIST 1.0 criteria) of 73%, 60% and 25% in patients who received prior TKIs, mTOR inhibitors or both respectively. The most common grade 3/4 toxicities of pazopanib included diarrhea, fatigue and hypertension, and drug dosage reductions were needed in the 21% of enrolled patients. More recently, pazopanib has been investigated in the multi-center, phase 2 Alliance A021202 study [34]. The trial randomized 171 patients with low-to-intermediate, progressive, extrapancreatic NETs to receive pazopanib or placebo. After a median follow-up of 31 months, a median PFS of 12 and 8 months was recorded in patients treated with pazopanib or placebo respectively (HR: 0.53; *p* = 0.0005). The rate of grade 3 or worse toxicities deemed to be related to pazopanib was 61%, with hypertension, fatigue, diarrhea and transaminases elevation reported to be the most common adverse events. Table 2 provides an overview of ongoing clinical trials of TKIs under clinical scrutiny in patients with NET.

## 4. Immunotherapeutic Agents

In recent years, multiple investigations have been carried out to characterize the immune microenvironment of NETs [35]. Immune cells including B and T cells, NK cells, mast cells, macrophages and dendritic cells have been reported to infiltrate NETs. Overall, the extent of tumor infiltration by immune cells appears to be higher in panNETs as compared with midgut NETs and higher in NECs compared to well-differentiated tumors [24], consistent with the mutational burden of these distinct tumor entities. The expression of programed death-ligand 1 (PD-L1) and programmed death 1 (PD-1) appears quite heterogeneous across different studies.

Multiple phase 2 trials have recently investigated single-agent or combination therapy with immune checkpoint inhibitors in patients with NETs or NECs, but the evidence of efficacy is inconsistent across studies (Table 3). The multi-cohort, phase 1b KEYNOTE-028 basket trial [36] evaluated the safety and efficacy of the PD-1 inhibitor pembrolizumab in patients with PD-L1-positive, advanced tumors including 41 patients with heavily pretreated, well- or moderately differentiated NETs. Overall, the ORR was 10%, with response durations ranging between 6.9 and 17.6 months. Seventy-one percent of patients experienced stable disease by RECIST 1.1. criteria. The subsequent phase 2 KEYNOTE-158 study [37] investigated pembrolizumab 200 mg every 3 weeks in 107 patients with well-differentiated, progressive NETs arising in the lung, appendix, small intestine, colon, rectum or pancreas. At study entry, 40% of patients had received at least 3 prior lines of treatment, while 16% of them had PD-L1-positive tumors. After a median follow-up of 24 months, the ORR by independent central review was 3.7% with 3 and 1 partial responses recorded in patients with pancreatic and rectal NETs respectively. All responding patients had PD-L1-negative tumors. The median PFS was 4.1 months, while the median overall survival (OS) was 24.2 months. Grade 3 or worse treatment-related adverse events occurred in the 21.5% of patients. The role of the tumor mutational burden (TMB) in predicting the efficacy of immunotherapy has been recently analyzed across the different cohorts included in the KEYNOTE-158 basket study. Among 87/107 NET patients with available TMB evaluation, 82 had a low TMB while 5 had a high TMB. Two objective responses (40%) were recorded in the group of patients with TMB-high tumors, whereas only one response out of 82 (1.2%) was reported in the TMB-low group [38]. Single-agent pembrolizumab has been also tested in two independent phase 2 trials that cumulatively enrolled 29 patients with high-grade neuroendocrine neoplasms who progressed on prior platinum-based chemotherapy [39]. The ORR was 3.4%, with stable disease in 20.7% of cases. The median PFS was 8.9 weeks, with no significant differences between the PD-L1 positive and negative groups.

Spartalizumab, a humanized mAb against PD-1, was investigated in 116 patients with progressive, non-functioning, well or poorly differentiated neuroendocrine neoplasms [40]. The ORR, assessed per RECIST 1.1 criteria by independent central radiology review, was 7.4% in well-differentiated NETs of GEP or pulmonary origin and 4.8% in GEP-NECs. Of interest, the subgroup of patients with lung NETs exhibited an ORR of 20%.

The PD-L1 inhibitor avelumab was tested in a phase 2 study [41] of 29 patients with G3 NETs or NECs of any origin excluding small cell lung cancer and Merkel cell carcinoma progressing after first-line chemotherapy. After 8 weeks of treatment, the DCR (partial response + stable disease according to irRECIST criteria) was 32%, with a mean duration of response among responders of 20 weeks. Treatment-related adverse events were mainly mild or moderate and occurred in the 38% of the cohort.

The dual blockade of PD-1 and cytotoxic T-lymphocyte antigen 4 (CTLA-4) was recently investigated in patients with NET. The phase 2 DART SWOG 1609 basket trial [42] tested ipilimumab 1 mg/kg every 6 weeks in combination with nivolumab 240 mg every 2 weeks in 32 patients with any grade, extra-pancreatic neuroendocrine neoplasms. Overall, the ORR was 25%, with one complete response and seven partial responses. All objective responses were recorded among patients with high-grade tumors, and in this subgroup of patients the ORR was 44% vs. 0% in patients with low-to-intermediate tumors. In the overall cohort, the 6-month PFS was 31%, and median OS was 11 months. In another phase 2 trial [43], 29 patients with any grade, advanced NETs received ipilimumab at 1 mg/kg every three weeks for four doses and nivolumab at 3 mg/kg, followed by nivolumab 3 mg/kg every two weeks for up to 96 weeks. At the time of data cut-off, the ORR was 24%, with objective responses observed in the 43% and 33% of patients with panNETs and atypical pulmonary carcinoids respectively. The median PFS was 4.8 months, with a median OS of 14.8 months. Grade 3 or worse immune-related toxicities were reported in the 34% of patients. The multi-cohort, phase 2 DUNE trial [44] has investigated the efficacy of PD-L1 and CTLA-4 blockade by durvalumab and tremelimumab in patients with progressive NETs of GEP and lung origin. A total of 123 patients were enrolled in four different cohorts: typical/atypical pulmonary carcinoids, low-to-intermediate grade gastrointestinal NETs, low-to-intermediate grade panNETs, and high-grade GEP neuroendocrine neoplasms. After a median follow-up of 10.8 months, the ORR by iRECIST criteria was 7.4%, 0%, 6.3% and 9.1% in the four cohorts respectively. Most frequent grade 3 or worse treatment-related adverse events were liver toxicity, diarrhea, fatigue and vomiting.

## 5. Future Perspectives

The expression of SSTRs on the surface of NET cells is presently targeted by both cold and radiolabeled somatostatin analogs, but innovative strategies for SSTR therapeutic targeting are being developed. Given that SSTR agonists are rapidly internalized upon binding to the receptor, SSTR agonists linked to a toxic payload have the potential to exert cytotoxic effects against NETs. PEN-221 is a recently developed molecule obtained through the process of conjugation of octreotate with the microtubule-damaging agent DM1. The maytansinoid conjugate has shown potent antitumor activity in preclinical studies [45], and a phase 1/2 clinical trial is currently investigating the safety and efficacy of the drug in patients with advanced, progressive, SSTR2-expressing NETs or NECs. Based on preliminary findings obtained from the first 21 patients enrolled in the study, PEN-221 has a favorable toxicity profile, with fatigue, nausea, diarrhea, vomiting, abdominal pain and decreased appetite as the most frequent treatment-emergent adverse events. Among the 15 patients evaluable for response, 11 had stable disease after 9 weeks from treatment initiation [46].

Bispecific antibodies against SSTRs constitute another possible strategy to target NETs through the redirection of T cell cytotoxicity against the tumor. A bispecific antibody targeting SSTR2 and CD3 (XmAb18087) has been recently developed [47], and its clinical testing is currently underway (NCT03411915). No objective responses were observed in the 14 patients with well-differentiated GEP- or pulmonary NETs evaluable according to RECIST 1.1 criteria [48].

CAR T cells directed against SSTR-expressing NET cells are under preclinical development. Preliminary evidence of their antitumor activity in vitro and in vivo has been recently reported [49].

New therapeutic targets are being intensively explored in patients with NETs. Given the role of the NTRK signaling pathway in the tumorigenesis, proliferation, and invasiveness of NETs, a phase 1, first-in-human study of the ROS1/NTRK inhibitor taletrectinib has been recently completed [50]. Among the 12 NET patients enrolled, one partial response was observed. The median PFS was 10.2 months, while diarrhea, nausea and vomiting were the most frequent treatment-emergent adverse events. Based on the preclinical evidence that cyclin-dependent kinase (CDK) inhibition has antitumor activity against NETs, a phase 2 trial [51] has recently assessed the safety and efficacy of the CDK4/6 inhibitor palbociclib in 21 patients with advanced, low-to-intermediate grade panNETs. After a median follow-up of 12.4 months, no objective responses were recorded, and median PFS was only 2.6 months.

Oncolytic viruses are increasingly used as an innovative form of cancer immunotherapy, and evidence of their antitumor activity has been already reported in patients with melanoma or head and neck cancer. The oncolytic adenovirus AdVince has been designed to use the gene promoter from human chromogranin A for selective replication in neuroendocrine cells, and preclinical studies have demonstrated its antitumor activity against NET cells. A phase 1/2 study is currently evaluating AdVince in patients with liver-dominant NETs of GEP or lung origin (NCT02749331).

## 6. Conclusions

Our understanding of the molecular events driving NET initiation and progression has improved substantially over the last two decades, and the treatment landscape of these malignancies has expanded accordingly, leading to prolonged patient survival. Among promising investigational treatments, the TKI surufatinib is in the most advanced stage of clinical development, and its use might be approved in patients with pancreatic and extra-pancreatic NETs in the next few years. While checkpoint inhibitor monotherapy appears to have limited antitumor activity, future clinical trials testing the dual blockade of PD-1 and CTLA-4 in patients with NECs may be of interest. In this context, whether combinations of immunotherapy/chemotherapy or immunotherapy/PRRT may show efficacy is currently unknown, as it is unclear whether defined treatment sequences (i.e., chemotherapy followed by immunotherapy or PRRT followed by immunotherapy) might improve outcomes by exploiting the abscopal effect. Encouraging results have been obtained in early phase clinical trials investigating the next generation of radiopeptides, and future studies of alpha-emitting agents or radiolabeled SSTR antagonists have the potential to further advance PRRT strategies.

## Figures and Tables

**Table 1:** Clinical trials of PRRT with alpha-emitters or somatostatin antagonists in patients with NETs.

Therapeutic Agent	Dosage and Schedule	Patient Population	Number of Patients Enrolled	Objective Responses * (RECIST 1.1)	Reference
**^225^** **Ac-DOTATATE**	Systemic infusion every 8 weeks (100 kBq/kg of body weight)	Advanced GEP-NETs stable or progressing on ^177^Lu-DOTATATE	32	ORR: 62% (62% PR; 38% SD)	[17]
**^213^** **Bi-DOTATOC**	Intra-arterial or systemic infusion every 8 weeks (first cycle: 1GBq; second cycle: 1.5 GBq; third cycle: 2–4 GBq; fourth cycle: as available from the generator)	Advanced NETs with liver metastases progressing on ^90^Y/^177^Lu-DOTATOC therapy	7	ORR: 50% (17% CR; 33% PR; 50% SD)	[18]
**^212^** **Pb-DOTAMTATE**	Systemic infusion every 8 weeks (3+3 dose escalation design)	Advanced SSTR^+^ NETs	50	ORR at highest dose cohort: 83% (1 CR; 5 PR)	[20]
**^177^** **Lu-DOTA-JR11**	Systemic infusion every 12 weeks (cumulative absorbed bone marrow dose up to 1 Gy)	Advanced, well-differentiated, SSTR^+^ NETs	20	ORR: 45% (5% CR; 40% PR)	[22]

* Among evaluable patients. NET: neuroendocrine tumor; RECIST: Response Evaluation Criteria in Solid Tumors; ORR: objective response rate; SD: stable disease; CR: complete response; PR: partial response; SSTR^+^: somatostatin receptor positive; PRRT: peptide receptor radionuclide therapy.

**Table 2:** Ongoing clinical trials of TKIs in patients with NETs.

Therapeutic Regimen	Molecular Target(s)	Clinical Phase	Patient Population	Sample Size	Primary Outcome	Identifier
**Cabozantinib**	c-MET, VEGFR2, AXL, KIT, TIE2, FLT3, RET	III	Advanced progressive NETs	395	PFS	NCT03375320
**Axitinib + Octreotide LAR vs. Placebo + Octreotide LAR**	VEGFR 1-3	II/III	Advanced, progressive, G1/G2 NETs of extra-pancreatic origin	255	PFS	NCT01744249
**Lenvatinib + Everolimus**	VEGFR 1-3, FGFR 1-4, IT, RET, PDGFR-alpha	II	Advanced progressive carcinoid tumors	32	ORR	NCT03950609
**Nintedanib**	VEGFR 1-3, PDGFR-α and -β, FGFR 1-3, FLT3, SRC	II	Advanced, G1/G2 NETs of extra-pancreatic origin	30	PFS	NCT02399215
**Famitinib**	c-KIT, VEGFR2-3, PDGFR, FLT1, FLT3	II	Advanced, G1/G2 GEP-NETs	53	ORR	NCT01994213
**Regorafenib**	VEGFR 1-3, PDGFRβ, KIT, RET, RAF-1	II	Advanced, progressive carcinoid or panNET	48	PFS	NCT02259725
**Anlotinib**	VEGFR2/3, FGFR1-4, PDGFR-α and -β, c-KIT, RET	II	G3 advanced GEP-NETs	60	PFS	NCT03457844
**Pazopanib + temozolomide**	VEGFR 1-3, PDGFR-α and -β, c-KIT	I/II	Advanced panNETs	29	MTD	NCT01465659
**Evofosfamide (TH-302) + Sunitinib**	DNA + VEGFR-1-3, PDGFR-α and -β, c-KIT, FLT-3, CSF1R	II	Advanced, G1/G2, treatment-naïve panNETs	43	ORR	NCT02402062

Abbreviations: AXL: AXL receptor tyrosine kinase; c-KIT: V-Kit Hardy-Zuckerman 4 feline sarcoma viral oncogene homolog; c-MET: MET proto-oncogene, receptor tyrosine kinase; CSF1R: colony stimulating factor 1 receptor; GEP: gastroenteropancreatic; FGFR: fibroblast growth factor receptor; FLT3: Fms related tyrosine kinase 3; MTD: maximum tolerated dose; NET: neuroendocrine tumor; ORR: objective response rate; panNET: pancreatic neuroendocrine tumor; PDGFR: platelet-derived growth factor receptor; PFS: progression-free survival; RAF: v-raf murine sarcoma viral oncogene homolog B1; RET: Ret proto-oncogene; SRC: SRC proto-oncogene, non-receptor tyrosine kinase; TIE2: tyrosine kinase with immunoglobulin and epidermal growth factor homology domains 2; VEGFR: vascular endothelial growth factor receptor.

**Table 3:** Clinical trials of immunotherapy in patients with NETs.

Therapeutic Agent	Dosage and Schedule	Patient Population	Number of Patients	Objective Responses (RECIST 1.1)	Reference
**Pembrolizumab**	10 mg/kg every 2 weeks	Advanced PD-L1^+^ carcinoids or panNETs	41	ORR: 12% (carcinoids); 6.3% (panNETs)	[36]
**Pembrolizumab**	200 mg every 3 weeks	Advanced, well-differentiated NETs	107	ORR: 3.7%	[37]
**Pembrolizumab**	200 mg every 3 weeks	Advanced G3 NETs/NECs (Ki-67 > 20%) progressing on platinum-based chemotherapy	29	ORR: 3.4%	[39]
**Spartalizumab**	400 mg every 4 weeks	Advanced thoracic/GEP-NETs and GEP-NECs	116	ORR: 7.4% (NETs); 4.8% (NECs)	[40]
**Avelumab**	10 mg/kg every 2 weeks	Advanced G3 NECs	29	ORR: 6.9% (by irRECIST criteria)	[41]
**Ipilimumab and Nivolumab**	Ipilimumab 1 mg/kg every 6 weeks; Nivolumab 240 mg every 2 weeks	Advanced, any grade NETs (excluding panNETs)	32	ORR: 25%	[42]
**Ipilimumab and Nivolumab**	Ipilimumab 1 mg/Kg every 3 weeks for four doses and Nivolumab 3 mg/Kg, followed by Nivolumab 3 mg/Kg every 2 weeks for up to 96 weeks	Advanced, any grade NETs	29	ORR: 24%	[43]
**Durvalumab and Tremelimumab**	Durvalumab 1500 mg every 4 weeks for 12 months, and Tremelimumab 75 mg every 4 weeks up to 4 doses/cycles	Cohort 1: well-moderately differentiated lung NETs Cohort 2: G1/G2 gastrointestinal NETs; Cohort 3: G1/2 panNETs Cohort 4: G3 GEP-NENs	126	ORR: 7.4% (cohort 1); 0% (cohort 2); 6.3% (cohort 3); 9.1% (cohort 4) (by irRECIST criteria)	[44]

+: positive; irRECIST: immune-related RECIST; NET: neuroendocrine tumor; ORR: objective response rate; PD: progressive disease; GEP: gastroenteropancreatic; NEC: neuroendocrine carcinoma; NENs: neuroendocrine neoplasms.
